# Assessing land-use changes and carbon storage: a case study of the Jialing River Basin, China

**DOI:** 10.1038/s41598-024-66742-2

**Published:** 2024-07-10

**Authors:** Shuai Yang, Liqin Li, Renhuan Zhu, Chao Luo, Xiong Lu, Mili Sun, Benchuan Xu

**Affiliations:** 1 Publicity and United Front Work Department, Nanchong Vocational College of Culture and Tourism, Nanchong, 637400 China; 2 Hotel Department, Nanchong Vocational College of Culture and Tourism, Nanchong, 637400 China; 3 CAS Key Laboratory of Mountain Ecological Restoration and Bioresource Utilization and Ecological Restoration Biodiversity Conservation Key Laboratory of Sichuan Province, Chengdu Institute of Biology, Chinese Academy of Sciences, Chengdu, 610041 China; 4 University of Chinese Academy of Sciences, Beijing, 100049 China; 5 College-Locality Cooperation and Training Center, Nanchong Vocational College of Culture and Tourism, Nanchong, 637400 China; 6 Department of Logistics, Nanchong Vocational College of Culture and Tourism, Nanchong, 637400 China; 7 Department of General Education, Nanchong Vocational College of Culture and Tourism, Nanchong, 637400 China

**Keywords:** Land-use change, PLUS model, InVEST model, Carbon storage, Jialing River Basin, Ecology, Environmental sciences

## Abstract

Land-use change is the main driver of carbon storage change in terrestrial ecosystems. Currently, domestic and international studies mainly focus on the impact of carbon storage changes on climate, while studies on the impact of land-use changes on carbon storage in complex terrestrial ecosystems are few. The Jialing River Basin (JRB), with a total area of ~ 160,000 km^2^, diverse topography, and elevation differences exceeding 5 km, is an ideal case for understanding the complex interactions between land-use change and carbon storage dynamics. Taking the JRB as our study area, we analyzed land-use changes from 2000 to 2020. Subsequently, we simulated land-use patterns for business-as-usual (BAU), cropland protection (CP), and ecological priority (EP) scenarios in 2035 using the PLUS model. Additionally, we assessed carbon storage using the InVEST model. This approach helps us to accurately understand the carbon change processes in regional complex terrestrial ecosystems and to formulate scientifically informed land-use policies. The results revealed the following: (1) Cropland was the most dominant land-use type (LUT) in the region, and it was the only LUT experiencing net reduction, with 92.22% of newly designated construction land originating from cropland. (2) In the JRB, total carbon storage steadily decreased after 2005, with significant spatial heterogeneity. This pattern was marked by higher carbon storage levels in the north and lower levels in the south, with a distinct demarcation line. The conversion of cropland to construction land is the main factor driving the reduction in carbon storage. (3) Compared with the BAU and EP scenarios, the CP scenario demonstrated a smaller reduction in cropland area, a smaller addition to construction land area, and a lower depletion in the JRB total carbon storage from 2020 to 2035. This study demonstrates the effectiveness of the PLUS and InVEST models in analyzing complex ecosystems and offers data support for quantitatively assessing regional ecosystem services. Strict adherence to the cropland replenishment task mandated by the Chinese government is crucial to increase cropland areas in the JRB and consequently enhance the carbon sequestration capacity of its ecosystem. Such efforts are vital for ensuring the food and ecological security of the JRB, particularly in the pursuit of the “dual-carbon” objective.

## Introduction

Land, as the primary arena for human activities, has undergone drastic changes owing to accelerating socioeconomic development, urbanization processes, and natural transformations by human beings^1,2^. Land-use change forms the foundation of the study of carbon storage in terrestrial ecosystems, as it directly impacts the original pattern, process, function, and structure of terrestrial ecosystems, thereby altering their carbon storage^3,4^. This directly affects climate change predictions, greenhouse gas emissions, and reduction efforts^4^. In the context of the overall spatial planning of the national territory, basins are open and composite systems and the basic unit of a complete ecosystem; moreover, the natural, economic, social, and cultural elements within basins are closely interconnected. Furthermore, basins are characterized by diverse natural landscapes, clear hierarchical structures, and holistic characteristics^2^. Taking a basin as a research area breaks traditional administrative boundaries. This approach allows for seeking optimal solutions for basin development that balance food production, ecology, environment, and other objectives, contributing to national ecological security construction^5^. Therefore, analyzing land-use changes in basins, predicting future land-use patterns, and quantitatively assessing ecosystem carbon storage are essential for achieving balanced development with multiple objectives in regions characterized by basins^6^.

Land-use simulation models are essential tools for studying future land-use dynamics. These models include quantitative models such as Markov^7^, linear prediction^8^, system dynamics^9^, gray prediction^10^ models, and spatial models such as patch-generating land use simulation (PLUS)^11^, future land use simulation (FLUS)^12^, cellular automaton (CA)^7^, and conversion of land use and its effects at small regional extent (CLUE-S)^13^. The PLUS model compensates for the shortcomings of the CA and CLUE-S models by effectively identifying the driving factors of land-use change and capturing the evolutionary patterns of various patch types. The model enhances the roulette wheel competition process and adaptive inertia within the FLUS model. Moreover, the integrated random forest algorithm of the model can effectively handle spatial autocorrelation and multicollinearity among the driving factors^11,14^. Consequently, the PLUS model can more accurately simulate various future land use scenarios and is extensively utilized^11,15^. The Markov model is widely used to predict future land-use demand by calculating the conversion probability between different land-use types (LUTs) over time^16–18^. Among all carbon storage assessment methods, the ecosystem functional state assessment and analysis model represented by Integrated Valuation of Ecosystem Service and Tradeoffs (InVEST) is highly regarded by scholars owing to the easy access to driving data, simple and convenient operation, high precision in quantitative assessment, and clear spatial representation of the assessment process and results^19,20^. The integration of PLUS, Markov, and InVEST models can enable the optimization of spatial layout, the quantitative structure of regional land-use, and the exploration of future carbon storage changing rules in terrestrial ecosystems.

The Jialing River Basin (JRB) connects the Guanzhong Plain urban agglomeration and the Lanxi urban agglomeration in the north and the Chengdu–Chongqing urban agglomeration in the south. It holds a crucial position in the Yangtze River Economic Belt and the Silk Road Economic Belt. As one of China’s most important grain-producing regions, the JRB is densely cultivated. The JRB is an essential part of the ecological barrier, a significant water source region, and a key area for biodiversity protection in the upper reaches of the Yangtze River in China. However, it is significantly affected by economic development and urbanization under the national macro-policy, leading to drastic land-use changes in the JRB. These changes bring about a series of challenges to food production and ecological security^21–23^. Carbon storage in terrestrial ecosystems plays a crucial role as carbon sinks. However, changes in carbon storage are primarily influenced by LUT conversion. The carbon sequestration function of terrestrial complex ecosystems has been consistently strengthened through the regulation of LUT conversion, which will aid China in realizing its “dual-carbon” goals. Studying the JRB land-use changes and their impact on carbon storage is vital. Although there have been numerous studies on the effects of land-use/cover changes or human activities on hydrological conditions^24^, soil erosion^25^, biodiversity^26^, and environmental pollution^23^ in the JRB, there is a lack of up-to-date studies on historical land-use or carbon storage changes and future land-use simulations or carbon storage assessments.

With the JRB as our research area, our objectives are as follows: (1) reveal the changing patterns of land use at the basin scale; (2) predict land-use status under business-as-usual (BAU), cropland protection (CP), and ecological priority (EP) scenarios in 2035 using the PLUS and Markov models; (3) assess carbon storage from 2000 to 2020 and in 2035 under different scenarios using the InVEST model; and (4) investigate carbon storage distribution and aggregation characteristics from 2000 to 2020 through global spatial autocorrelation analysis (Moran’s I), clustering and outlier analysis (Anselin Local Moran’s I), and cold-hotspot analysis (Getis-Ord G_i_*). This research aims to provide essential data support for achieving sustainable development in the JRB. Our study revealed that cropland, being the most prominent and the only LUT transferred out of the JRB, is in direct competition with construction land. To realize the coordinated ecological, social, and economic development of the JRB, it is imperative to implement CP policies, increase afforestation and grass-planting efforts, and strictly enforce urban development boundaries.

## Materials and methods

### Study area

**Figure 1:**
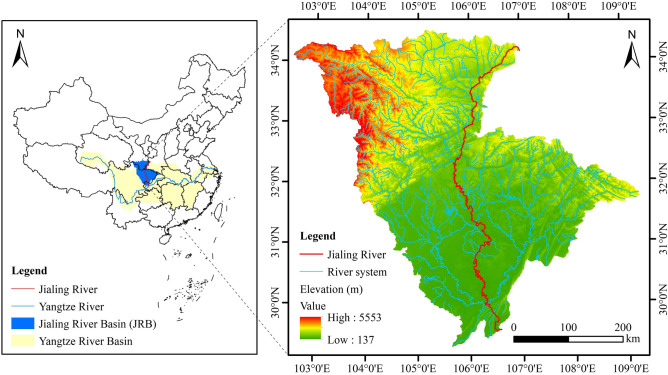
Location and terrain of the study area.

The Jialing River is a primary tributary of the upper reaches of the Yangtze River on the left bank, with a total length of 1,345 km. Its water system has a dendritic shape, and most of it flows through the eastern part of the Sichuan Basin, eventually joining the Yangtze River at Chaotianmen in the Yuzhong District of Chongqing Municipality^25,27^. Covering a total area of ~ 160,000 km^2^, the JRB (longitude 102° 31′ 51″–109° 16′ 34″, latitude 29° 17′ 29″–34° 31′ 44″) constitutes ~ 9% of the Yangtze River Basin. It spans Shaanxi, Gansu, Sichuan, and Chongqing (Fig. 1)^28^.

The JRB is situated in the transition zone from the Qinghai–Tibet Plateau to China’s second-tier terrain, characterized by complex and varied topography. It encompasses the plateau region, mountainous region, hilly region, and basin region, displaying distinct geographic zoning characteristics. The JRB terrain tilts roughly from northwest to southeast, exhibiting a significant gradient change. The elevation difference across the entire JRB exceeds 5 km, resulting in dramatic topographical variations. The river course aligns with the terrain, leading to an elevation difference in the river of ~ 2.3 km and an average drop ratio of 2.05‰^29^. The JRB traverses multiple climate zones, including the Tibetan Plateau, temperate monsoon, and subtropical monsoon regions. These climate zones exhibit distinct characteristics, with hot and rainy summers and warm and humid winters. The multi-year average daily maximum and minimum temperatures in the JRB are 19.4 °C and 4.3 °C, respectively^30^. Precipitation in the JRB follows a spatial distribution pattern that decreases from southeast to northwest. The multi-year average, maximum, and minimum precipitation levels are 935 mm, 1,283 mm, and 643 mm, respectively^28,29^.

### Data sources

#### Land-use data and its driving factors

The standard Chinese map with the approval number of GS(2024)0650 No. was obtained from the National Platform for Common GeoSpatial Information Services (https://www.tianditu.gov.cn/). Data for the Jialing River, Yangtze River, and their respective basins were provided by the National Cryosphere Desert Data Center (https://www.ncdc.ac.cn). Land-use data were obtained from the Resource and Environment Science and Data Center of the Chinese Academy of Sciences (https://www.resdc.cn). This dataset includes six primary land classes, such as cropland and forestland, and 24 secondary land classes, including paddy fields and dry land. Using ArcGIS 10.3 software, we cropped the data according to the vector range of the JRB and reclassified them into six LUTs: cropland, forestland, grassland, water, construction land, and unused land. Subsequently, we generated five land-use raster maps of the JRB for years 2000, 2005, 2010, 2015, and 2020. Drawing upon relevant studies^1,16,31,32^, we selected 19 driving factors encompassing both natural environmental and socioeconomic aspects. Among these, average annual temperature, average annual precipitation, total phosphorus, total potassium, total nitrogen, and soil organic matter were obtained from the National Tibetan Plateau Data Center (https://data.tpdc.ac.cn) and the National Earth System Science Data Center (https://www.geodata.cn). Digital elevation model (DEM) data were sourced from the Geospatial Data Cloud (https://www.gscloud.cn), while slope data were calculated from the DEM data using ArcGIS 10.3 software. Gross domestic product, population density, and nighttime lighting data were retrieved from the Resource and Environment Science and Data Center of the Chinese Academy of Sciences (https://www.resdc.cn). County (city and district) governmental location data were obtained from OpenStreetMap (https://www.openstreetmap.org), and data related to railways, Class I–IV roads, rivers, and settlements were acquired from the National Catalogue Service For Geographic Information (https://www.webmap.cn). Using ArcGIS 10.3 software, the projection coordinate system of the land-use data and data on the natural environment and socioeconomic factors were standardized to CGCS2000_GK_Zone_18. Subsequently, the data on roads, rivers, settlements, and county (city and district) governmental sites were subjected to Euclidean distance analysis. All data were converted into raster data (.tif) with a spatial resolution of 30 m × 30 m.

#### Carbon density data

According to the methodologies outlined by Li et al.^33^, Zhang et al.^34^, Wang et al.^35^, and Xiang et al.^36^ for determining carbon density, we collated four datasets on carbon density: Dataset 1 comprised experimentally determined carbon density data on the JRB and its neighboring regions. A dataset on carbon density in Chinese terrestrial ecosystems (2010s)^37^ obtained from the Institute of Geographic Sciences and Natural Resources Research of the Chinese Academy of Sciences was processed in ArcGIS 10.3 to extract carbon density measurements in the study area and its surroundings. In addition, we utilized carbon density data obtained by Xia et al.^38^ and Xia et al.^39^. Dataset 2 comprised carbon density data on the JRB, and it was collected from Zhang et al.^40^. Dataset 3 comprised carbon density data on the surrounding areas of the JRB, and it was collected from Xiang et al.^36^. The data were corrected for carbon density using the mean annual temperature and mean annual precipitation correction models^41^. Dataset 4 comprised carbon density data on the climatic zone of the study area, and it was collected from Liu et al.^42^. Finally, we analyzed the collected carbon density data to remove outliers, and then took the average of the carbon density of each component for each LUT as the carbon pool data for the InVEST model (Table 1).

**Table 1:** Carbon density data of various land-use types (LUTs) in the Jialing River Basin (JRB) (unit: t hm^−2^).

LUT	Aboveground	Belowground	Soil organic matter	Dead organic matter	Total
Cropland	4.94	3.09	92.42	0.62	101.07
Forestland	37.43	8.53	97.34	3.28	146.58
Grassland	1.21	4.44	201.84	0.69	208.18
Water	0.21	0	106.78	0	106.99
Construction land	0.16	0.30	45.48	0	45.94
Unused land	1.83	11.51	169.91	0.19	183.44

### Methods

**Figure 2:**
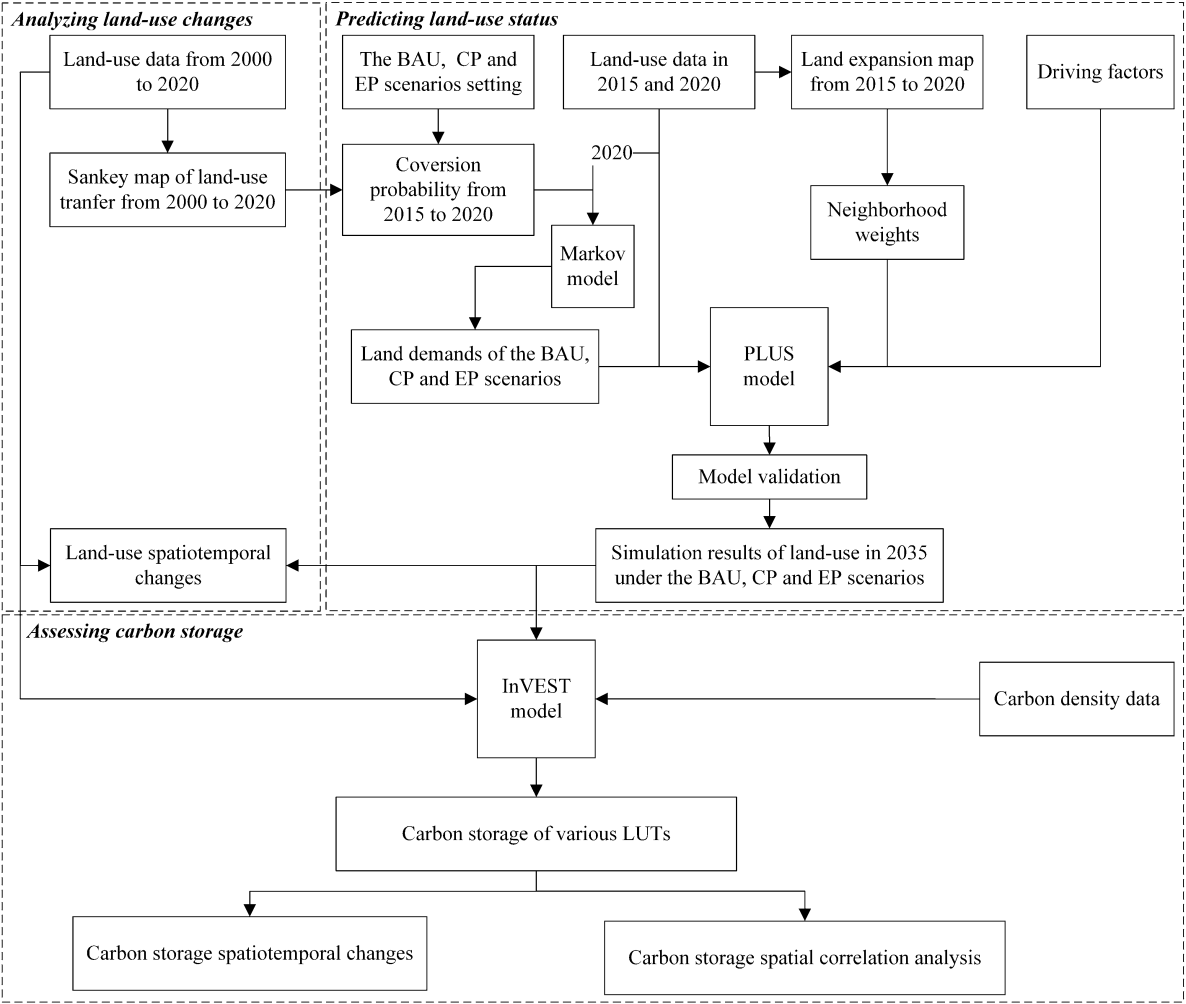
The research framework of this study.

The research flowchart of the simulation and assessment of land-use changes and carbon storage in this paper is shown in Fig. 2.

#### Multi-scenario setting

According to policy documents such as the Territorial Spatial Plan (2021–2035) of Shaanxi Province, Gansu Province, Sichuan Province, and Chongqing Municipality (municipalities directly under the central government) where the JRB is situated, we established the simulation timeframe for future land use in the JRB as the year 2035. The JRB, an important ecological reserve and grain-producing region in the upper reaches of the Yangtze River, predominantly consists of cropland, forestland, and grassland as its land use types. Drawing on the land use scenario simulation studies conducted by Zhang et al.^40^ within the JRB and by Yang et al.^43^ in the surrounding areas, we established the BAU, CP, and EP scenarios for the future land use of the JRB. The specifics of these scenarios are described as follows:

The BAU scenario was constructed based on land-use change ratios, socioeconomic factors, and natural environmental drivers from 2015 to 2020, without considering policy planning constraints. The Markov model was employed to predict the future demand for various LUTs, and the LUT demand served as a parameter for land-use demand in the PLUS model^2^. This scenario formed the basis for setting other scenarios.

The CP scenario was built upon the BAU scenario according to the setup parameters presented in Li et al.^19^, Wang et al.^44^, and Li et al.^45^. In this scenario, the Markov transfer probability matrix was adjusted. The conversion probability of cropland to construction land was reduced by 65% to rigorously enforce CP policies.

The EP scenario was developed based on the BAU scenario according to the setup parameters presented in Li et al.^16^, Wang et al.^44^, and Li et al.^45^. In this scenario, through the modification of the Markov transfer probability matrix, the conversion probabilities of forestland and grassland to construction land were 50% lower than that in the BAU scenario, while the conversion probability of water to construction land was 30% lower. Additionally, the ecological capacity of cropland was weaker than that of forestland. Therefore, the probability of cropland being converted into construction land was 25% lower than that in the BAU scenario.

#### Markov model

The Markov model, based on the Markov chain process, is a statistical method for predicting future probability, and it is characterized by the non-aftereffect property, stochasticity, discreteness, and stationarity^16,46^. It predicts future land-use change trends using initial state and transfer probability matrices^19^. The formula for the model is as follows:

1 $${S}_{t+1} = {S}_{t} \times {P}_{ij}$$

where ${S}_{t}$ and ${S}_{t+1}$ are the land-use states at $t$ and $t+1$, respectively; ${P}_{ij}$ is the transfer probability of LUT $i$ to $j$ during the study period.

To minimize errors generated by the Markov model in long time series, in this study, the LUT demand under each scenario was predicted sequentially at five-year intervals, that is, for 2025, 2030, and 2035.

### PLUS model, key parameter setting, model validation

The PLUS model enables the generation of land-use change simulations using raster data patches, allowing for the exploration of causal factors influencing various LUT changes and the simulation of changes at the patch level^11^. This model comprises two modules: a rule-mining framework based on a land expansion analysis strategy (LEAS) and a CA model based on multi-type random patch seeds (CARS)^11^. The LEAS module can extract and sample land-use expansion between two periods of land-use change, employing the random forest algorithm to mine and acquire development probabilities and the contribution ratios of drivers for various LUTs^47^. Under the constraints of development probabilities, the CARS module simulates the automatic generation of patches by combining randomly generated seeds and employing decreasing threshold mechanisms^48^.

Neighborhood weight setting

Neighborhood weight indicates the expansion capacity of various LUTs, with values ranging from 0 to 1^19^. This study determined the parameters for neighborhood weights for different scenarios according to the expansion area share of various LUTs^49^, combined with insights from related studies (see Table 2)^33,50^.

**Table 2:** Neighborhood weight. Business-as-usual (BAU), cropland protection (CP), and ecological priority (EP) scenarios.

Scenarios	Cropland	Forestland	Grassland	Water	Construction land	Unused land
BAU	0.25	0.04	0.02	0.07	0.95	0.01
CP	0.40	0.03	0.01	0.10	0.90	0.01
EP	0.30	0.15	0.10	0.10	0.90	0.01

Cost matrix setting

A cost matrix represents the conversion rules between various LUTs. A matrix value of 0 is assigned when one LUT cannot be converted into another, and a matrix value of 1 is assigned when the opposite is true. Table 3 presents the cost matrices for different scenarios.

**Table 3:** Cost matrix under different scenarios.

LUT	BAU scenario						CP scenario						EP scenario					
	A	B	C	D	E	F	A	B	C	D	E	F	A	B	C	D	E	F
A	1	1	1	1	1	1	1	0	0	0	0	0	1	1	1	1	1	1
B	1	1	1	1	1	1	1	1	1	1	1	1	0	1	1	0	0	0
C	1	1	1	1	1	1	1	1	1	1	1	1	0	1	1	0	0	0
D	1	1	1	1	1	1	1	1	1	1	1	1	1	1	1	1	0	1
E	0	0	0	0	1	0	0	0	0	0	1	0	0	0	0	0	1	0
F	1	1	1	1	1	1	1	1	1	1	1	1	1	1	1	1	1	1

Cropland (A), forestland (B), grassland (C), water (D), construction land (E), and unused land (F).

Restricted region setting

In different scenarios, the river water surface was transformed into a binary image, with 0 indicating the non-transformable region and 1 indicating the transformable region. These images were then input into the PLUS model as restricted factors.

PLUS model validation

To assess the accuracy of land-use simulation results, overall accuracy (OA), and the figure of merit (FoM) coefficient were employed. An OA approaching 1 indicates higher simulation accuracy, with an OA value of > 0.75 indicating a reliable simulation^48^, while a smaller FoM value indicates higher simulation accuracy^51^. The formula for FoM is as follows^52^:

2 $$FoM=\frac{Hits}{Misses+Hits+Wrong \,Hits+False \,Alarms}$$

where *Misses* is the area of error due to reference change simulated as persistence; *Hits* is the area correctly identified as change resulting from reference change; *Wrong Hits* is the area of error due to reference change simulated as change to the wrong category; *False Alarms* is the area of error due to reference persistence simulated as change.

Consequently, based on land-use data from 2015, the simulation data for 2020 generated by the PLUS model prediction were compared with the actual data. The results revealed an OA of 0.9961, and an FoM coefficient of 0.0232, indicating that the model can accurately simulate future land-use changes in the JRB.

#### Carbon storage

InVEST model

The carbon module of the InVEST model assumes that various LUTs correspond to total carbon density, which consists of belowground carbon density, aboveground carbon density, dead organic matter carbon density, and soil organic matter carbon density. The model considers the carbon density of various LUTs to be constant^53^. The calculation formula is as follows:

3 $${C}_{i} = {C}_{i-above} + {C}_{i-below} + {C}_{i-dead} + {C}_{i-soil}$$

4 $${C}_{i-total} = \sum_{i=1}^{n}{C}_{i}\times {S}_{i}$$

In formulas (3) and (4), $i$ denotes the *i*-th LUT; ${C}_{i}$ is the total carbon density of LUT *I*; ${C}_{i-above}$, ${C}_{i-below}$, ${C}_{i-dead}$, and ${C}_{i-soil}$ are the aboveground, belowground, dead organic matter, and soil organic matter carbon densities of LUT *I*, respectively; ${C}_{i-total}$ is the total carbon storage of LUT *I*; *n* is the total number of LUTs; and ${S}_{i}$ is the area of LUT *I*.

Spatial correlation analysis

Spatial autocorrelation is a common method used to test whether the attribute values of elements are spatially correlated and the degree of spatial relevance^49^. This measure helps identify the extent to which attributes are clustered or dispersed. According to previous studies^49,54,55^, to account for the actual conditions in the JRB and the complexity of data processing, a grid of 9 km × 9 km was generated, along with grid points within the study area, using ArcGIS 10.3. Carbon storage data were linked with the grid to obtain the carbon storage value for each grid point. At the grid scale, global autocorrelation results were derived by calculating the spatial autocorrelation Global Moran’s I index. Subsequently, outlier or clustering location maps (i.e., local indicators of spatial association [LISA] clustering maps) were generated through clustering and outlier analysis. Finally, cold-hotspot analysis was employed to study the spatial distribution pattern of high-value and low-value carbon storage clusters in the JRB, aiding in the detection of unusual events. Detailed formulas and additional information on spatial autocorrelation analysis can be found in the literature^56,57^.

## Results

### Land-use changes, 2000–2020

From 2000 to 2020, the JRB was primarily composed of cropland, forestland, and grassland, accounting for more than 43%, 32%, and 21% of the total basin area, respectively (Table 4). The remaining LUTs covered smaller areas, each accounting for less than 2% of the total basin area. Each LUT area underwent varying degrees of change, with the most significant change occurring in cropland areas, which continued to decrease, resulting in a total reduction of 1536.98 km^2^ or 2.16%. Conversely, the water and construction land areas continued to increase, with additional values of 107.24 and 1087.94 km^2^, representing increases of 6.77% and 66.40%, respectively. The areas of forestland, grassland, and unused land also generally increased, with additional values of 239.89, 49.10, and 52.81 km^2^, corresponding to increases of 0.46%, 0.14%, and 11.10%, respectively. These changes were primarily due to rapid urbanization in the JRB, leading to significant encroachment of construction land on cropland. Additionally, the national policy of “returning cropland to forest for grass” resulted in the conversion of cropland to forestland and grassland. Moreover, the state’s comprehensive promotion of ecological restoration and protection in the Yangtze River Basin played a crucial role in increasing the water area.

**Table 4:** Changes of each land-use type (LUT) in the JRB over time from 2000 to 2020.

LUT	2000		2005		2010		2015		2020		2000–2020	
	Area (km^2^)	Proportion (%)	Area (km^2^)	Proportion (%)	Area (km^2^)	Proportion (%)	Area (km^2^)	Proportion (%)	Area (km^2^)	Proportion (%)	Area change (km^2^)	Proportion change (%)
Cropland	71,303.86	44.23	70,743.84	43.88	70,567.54	43.77	69,984.20	43.41	69,766.88	43.27	− 1536.98	− 2.16
Forestland	51,598.51	32.00	51,789.23	32.12	51,936.31	32.21	51,893.82	32.19	51,838.40	32.15	239.89	0.46
Grassland	34,626.29	21.48	34,814.75	21.59	34,692.40	21.52	34,681.17	21.51	34,675.39	21.51	49.10	0.14
Water	1584.69	0.98	1587.35	0.98	1606.90	1.00	1674.21	1.04	1691.93	1.05	107.24	6.77
Construction land	1638.56	1.02	1815.62	1.13	1912.63	1.19	2464.25	1.53	2726.50	1.69	1087.94	66.40
Unused land	475.76	0.30	476.87	0.30	511.90	0.32	530.03	0.33	528.57	0.33	52.81	11.10

**Figure 3:**
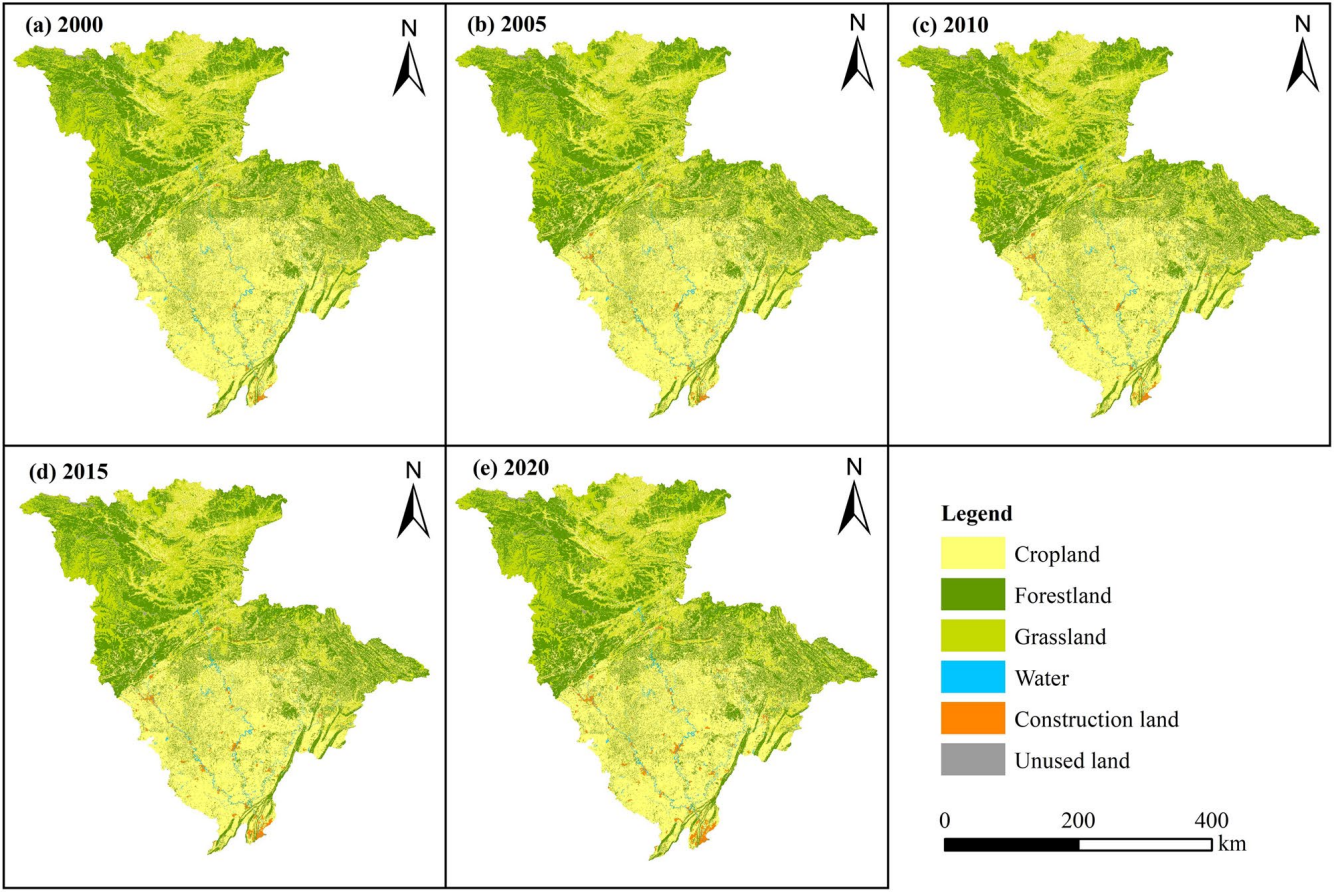
Land-use spatiotemporal distribution in the JRB from 2000 to 2020.

Cropland was mainly concentrated in the Sichuan Basin in the south of JRB, and it was also distributed in the eastern, northern, and central regions (Fig. 3). Forestland was predominantly situated in the mountainous areas surrounding the Sichuan Basin, as well as in the middle and upper reaches of the JRB. Grassland was mainly found in the middle and upper reaches of the JRB. Construction land was primarily located on both sides of the river in the middle and lower reaches of the JRB, while unused land was situated at higher elevations in the northwest of the basin.

**Figure 4:**
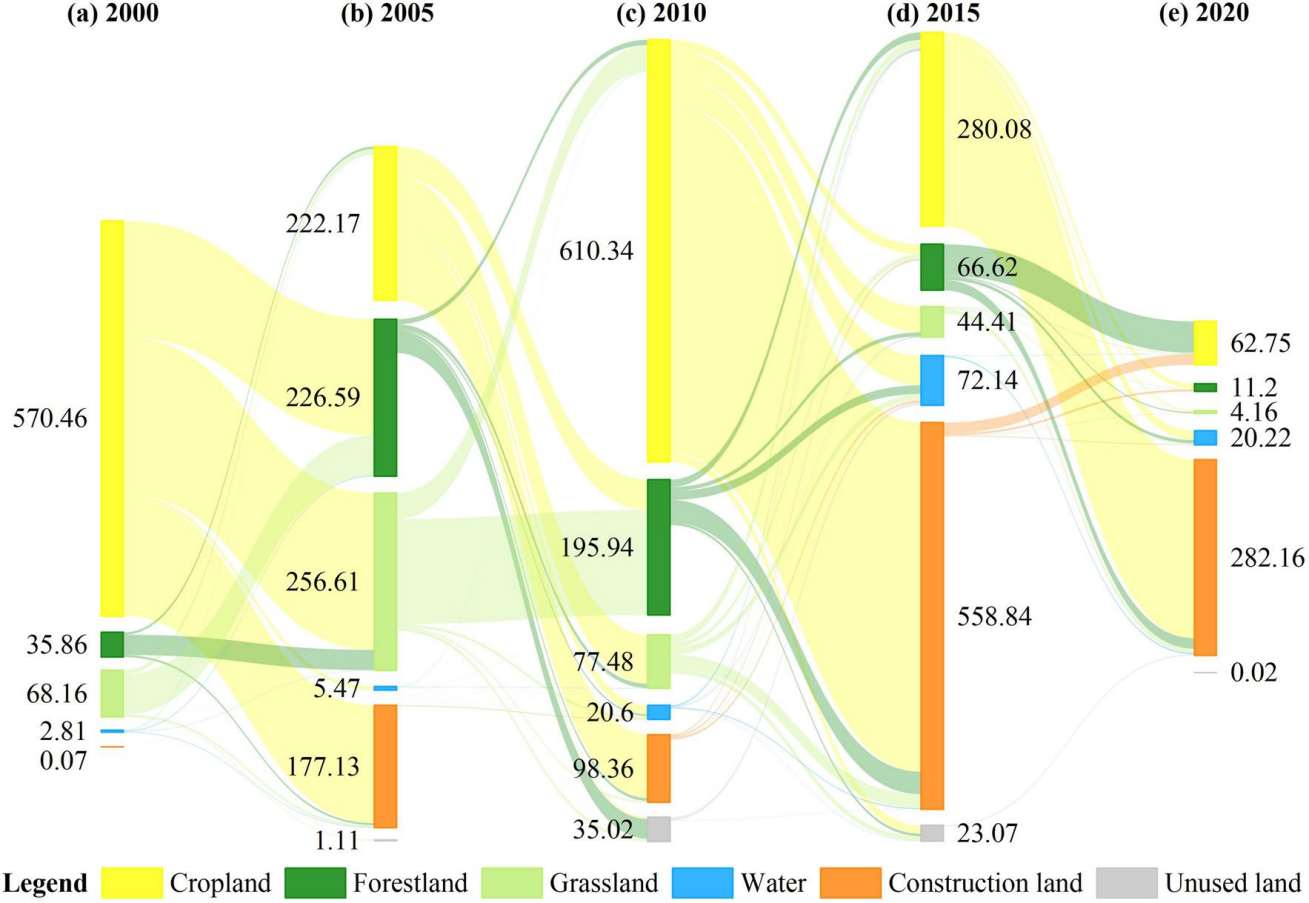
Sankey map of land-use transfer for the JRB, 2000–2020 (km^2^).

A total of 1673.27 km^2^ of cropland in the JRB underwent changes from 2000 to 2020, with 61.11% of cropland being converted into construction land, while 136.29 km^2^ was transformed into cropland (Fig. 4). Forest land experienced an addition of 451.75 km^2^ and a loss of 211.86 km^2^. Grassland experienced an addition of 377.70 km^2^ and a loss of 328.59 km^2^. Water area experienced an addition of 116.82 km^2^ and a loss of 9.58 km^2^. Construction land experienced an addition of 1108.84 km^2^, of which 92.22% was from cropland, and a loss of 20.91 km^2^. The net increase in the areas of forestland, grassland, water, and construction land accounted for 15.61%, 3.19%, 6.98%, and 70.78% of the total net increase in area, respectively. This indicates that the significant decrease in cropland area was primarily due to its conversion into construction land. The conversion of forestland and grassland into cropland also contributed to the decrease in cropland area.

### Carbon storage spatiotemporal changes, 2000–2020

Carbon storage in the JRB first increased and then decreased over the years from 2000 to 2020, reaching its peak in 2005 (Table 5). The carbon storage values in 2000, 2005, 2010, 2015, and 2020 (i.e., five-year intervals) were 2,231.06, 2,232.98, 2,232.10, 2,228.94, and 2,227.18 × 10^6^ t, respectively. This represents a total decrease of 3.88 × 10^6^ t, with an average decrease of 0.17%. Carbon storage changes differed among various LUTs. Cropland, forestland, and grassland were the most essential carbon pools in the JRB, each accounting for more than 30% of the total carbon storage. Together, they comprised over 98% of the total carbon storage, and their combined values continued to decrease over time. The combined highest and lowest carbon storage values of these three LUTs were 2,197.85 × 10^6^ (in 2000) and 2,186.85 × 10^6^ t (in 2020), representing 98.51% and 98.19% of the total, respectively. Cropland carbon storage decreased by 2.16% from 2000 to 2020, while water and construction land carbon storage increased by 6.77% and 66.40%, respectively, from 2000 to 2020. The carbon storage of forestland, grassland, and unused land initially increased and then decreased over the years from 2000 to 2020, with overall increases of 0.46%, 0.14%, and 11.10%, respectively.

**Table 5:** Changes in the carbon storage of each LUT in the JRB over time, 2000–2020.

LUT	2000		2005		2010		2015		2020		2000–2020	
	Carbon storage (10^6^ t)	Proportion (%)	Carbon storage (10^6^ t)	Proportion (%)	Carbon storage (10^6^ t)	Proportion (%)	Carbon storage (10^6^ t)	Proportion (%)	Carbon storage (10^6^ t)	Proportion (%)	Carbon storage change (10^6^ t)	Proportion change (%)
Cropland	720.67	32.30	715.01	32.02	713.23	31.95	707.33	31.73	705.13	31.66	− 15.53	− 2.16
Forestland	756.33	33.90	759.13	34.00	761.28	34.11	760.66	34.13	759.85	34.12	3.52	0.46
Grassland	720.85	32.31	724.77	32.46	722.23	32.36	721.99	32.39	721.87	32.41	1.02	0.14
Water	16.95	0.76	16.98	0.76	17.19	0.77	17.91	0.80	18.10	0.81	1.15	6.77
Construction land	7.53	0.34	8.34	0.37	8.79	0.39	11.32	0.51	12.53	0.56	5.00	66.40
Unused land	8.73	0.39	8.75	0.39	9.39	0.42	9.72	0.44	9.70	0.44	0.97	11.10
Total	2231.06	100.00	2232.98	100.00	2232.10	100.00	2228.94	100.00	2227.18	100.00	− 3.88	− 0.17

**Figure 5:**
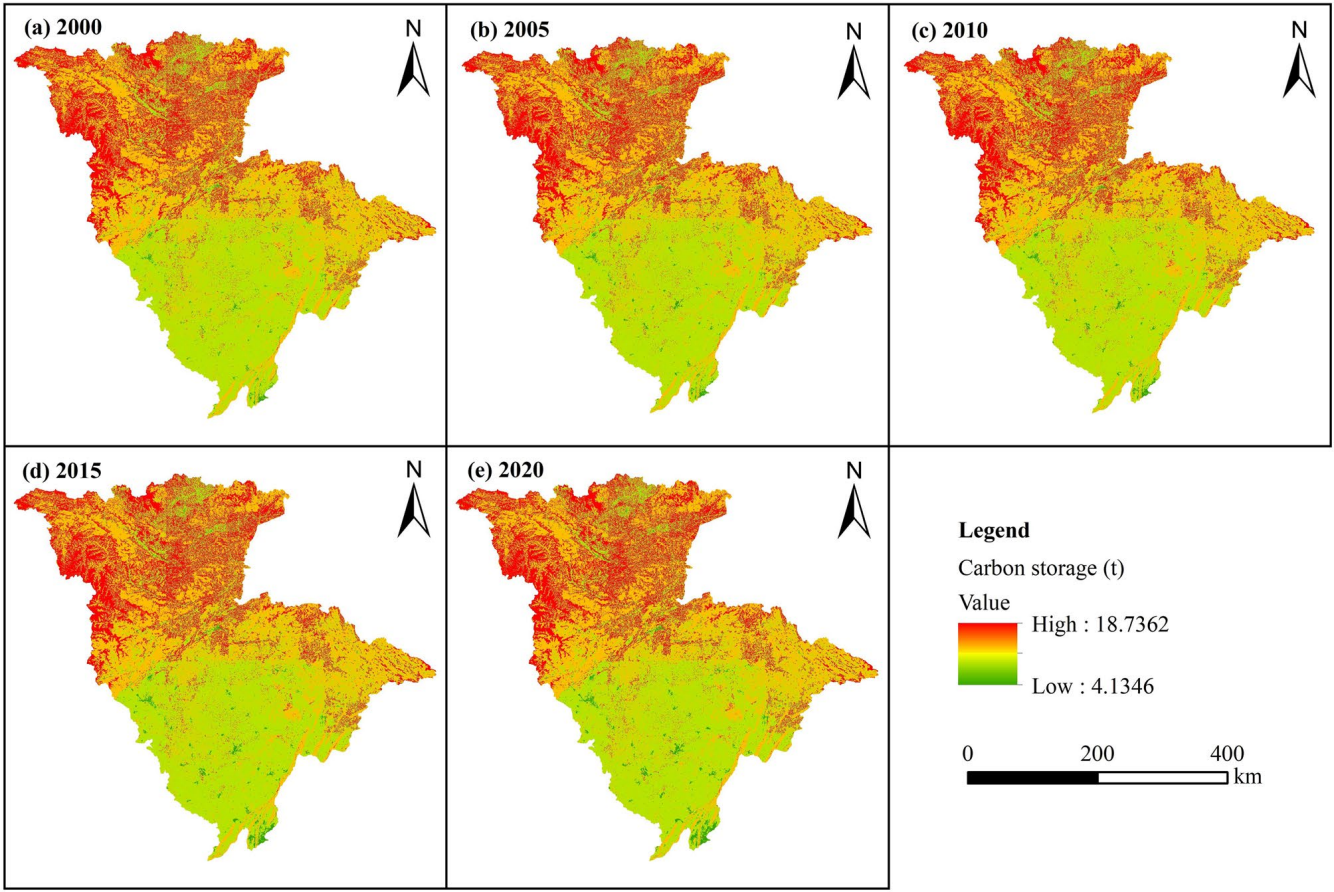
Spatiotemporal distribution of carbon storage in the JRB from 2000 to 2020.

The distribution of carbon storage was strongly correlated with LUT (Fig. 5), with higher carbon storage values in the north and lower values in the south. High-value regions were predominantly located in the northwestern and northern mountainous regions, while low-value regions were mostly situated in the hilly areas of the Sichuan Basin.

**Figure 6:**
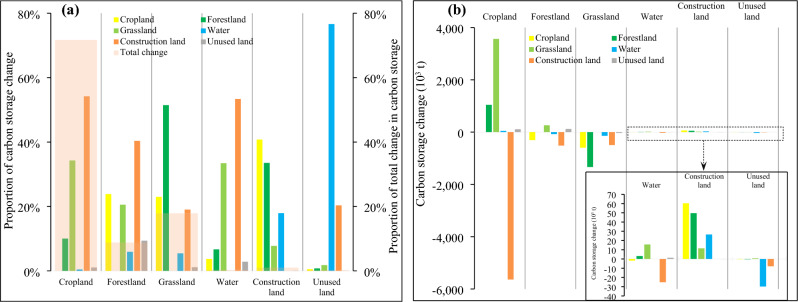
(**a**) Proportions of carbon storage variation and (**b**) carbon storage variations in the conversions of different LUTs in the JRB, 2000–2020.

Different LUT conversions impacted carbon storage owing to the effects of various LUT transfers and differences in carbon density (Fig. 6). Among these, the carbon storage changes in cropland, forestland, grassland, water, and construction land accounted for 71.70%, 8.79%, 17.90%, 0.32%, and 1.02% of the total changes, respectively. Carbon storage changes in cropland from 2000 to 2020 primarily reflected the conversion of cropland into construction land, grassland, and forestland. Carbon storage changes in forestland mainly resulted from the conversion of forestland into construction land, cropland, and grassland. Carbon storage changes in grassland were mainly driven by the conversion of grassland into forestland, cropland, and construction land. Carbon storage changes in water mainly stemmed from the conversion of water into construction land and grassland. Carbon storage changes in construction land were primarily due to conversions into cropland, forestland, and water.

### Carbon storage spatial correlation analysis from 2000 to 2020

Global spatial autocorrelation analysis of the JRB carbon storage was conducted to obtain the Global Moran’s I index for the five time points from 2000 to 2020 (Table 6). Under a 0.01 significance test level, the *Z*-score values for 2000, 2005, 2010, 2015, and 2020 were all greater than the test threshold value of 2.58, indicating a highly significant result. The Global Moran’s I index values of 0.9076, 0.9078, 0.9089, 0.9081, and 0.9094 for 2000, 2005, 2010, 2015, and 2020 were > 0, indicating that the spatial distribution of the JRB carbon storage exhibited apparent aggregation. The Global Moran’s I index gradually increased over time, reaching extremely high values in 2010 and 2020, suggesting that the spatial distribution of carbon storage in the JRB showed fluctuating but overall strengthening agglomeration trends.

**Table 6:** Global Moran’s I index of carbon storage in the JRB, 2000–2020.

Year	Moran’s I	Expectation Index	Variance	*Z*-score	*P*-value
2000	0.9076	− 0.0005	0.0001	81.9500	0.0000
2005	0.9078	− 0.0005	0.0001	81.9689	0.0000
2010	0.9089	− 0.0005	0.0001	82.0708	0.0000
2015	0.9081	− 0.0005	0.0001	81.9960	0.0000
2020	0.9094	− 0.0005	0.0001	82.1164	0.0000

**Figure 7:**
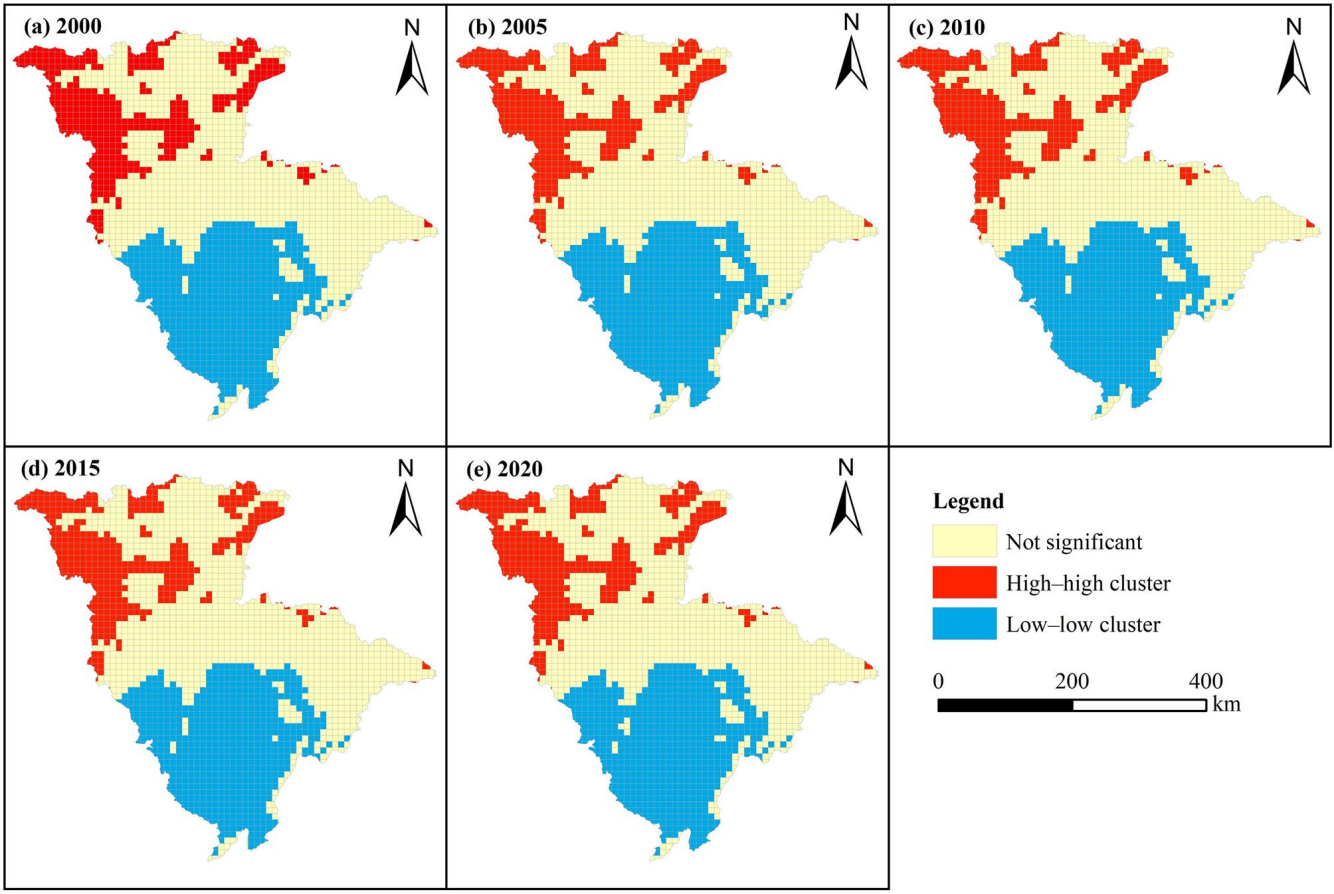
LISA agglomeration diagram of carbon storage in the JRB, 2000–2020.

To further analyze the spatial clustering patterns of carbon storage distribution in the JRB, a spatial LISA clustering map was constructed using local autocorrelation analysis (Fig. 7). The clustering of carbon storage was characterized by high levels in the north and low levels in the south. The “high–high” clustering region was relatively scattered, mainly distributed in JRB’s northwestern area. The “low–low” clustering region was more concentrated, mainly in JRB’s middle and lower reaches. Neither the “high–high” nor the “low–low” clustering regions changed significantly over time.

**Figure 8:**
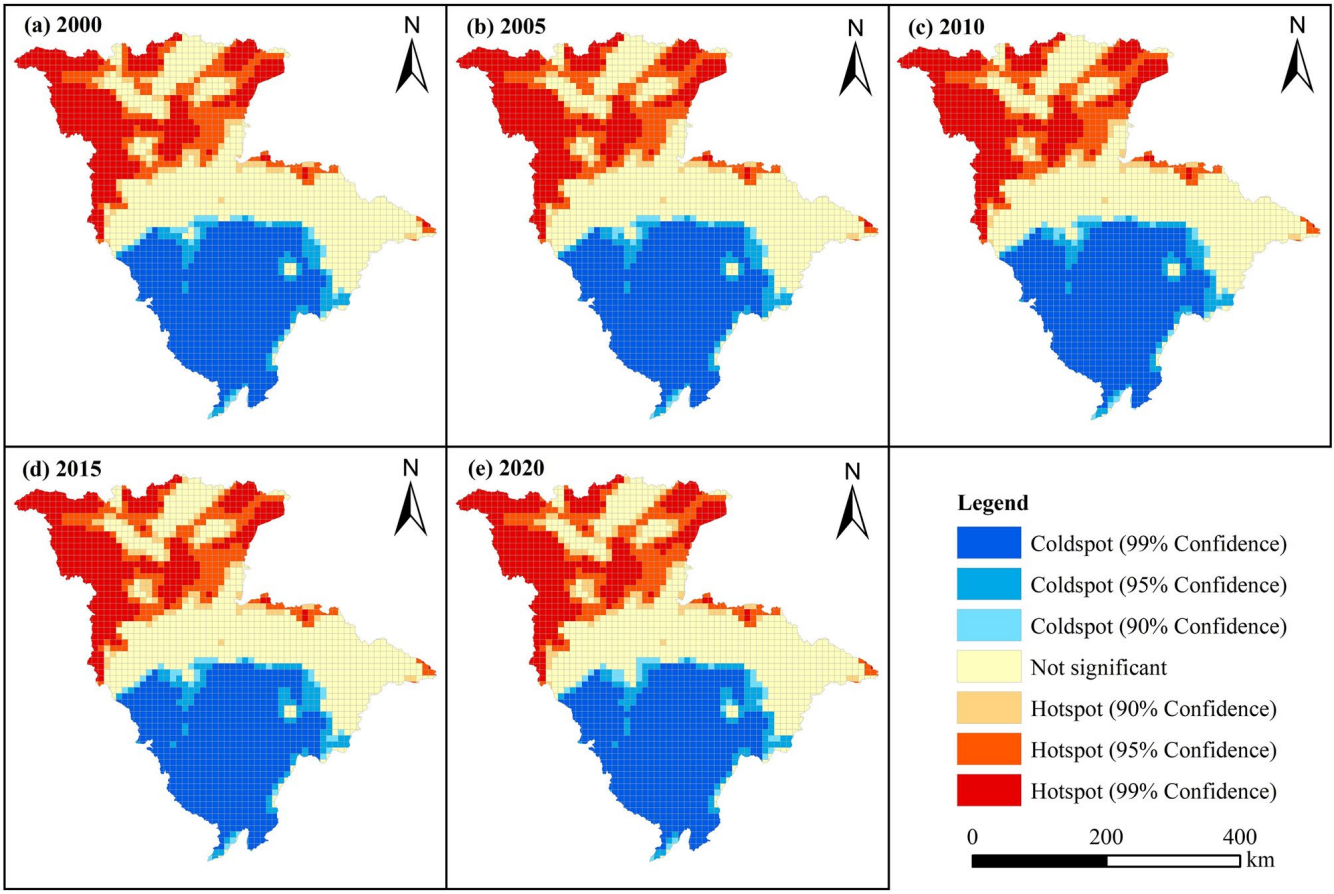
Cold-spot and hot-spot distribution of carbon storage in the JRB from 2000 to 2020.

To further investigate the spatial location and degree of high-value and low-value clusters of carbon storage in the JRB, a cold-hotspot analysis was conducted (Fig. 8). The results indicated that the cold spots and hot spots of carbon storage in the JRB were clustered with significant cold-hotspot effects and had a high degree of spatial differentiation. No transformation occurred between cold spots and hot spots. Carbon storage hot spots were distributed relatively scatteredly, mainly in JRB’s upper and northwestern regions. This distribution was primarily due to forestland and grassland dominating the region, with significant terrain undulations, high elevation, relatively low temperature, and precipitation. The state vigorously implemented ecosystem protection and restoration measures in the region, resulting in a rich and high coverage of vegetation types. Carbon storage cold spots were concentrated and stable, distributed in JRB’s middle and lower reaches in a centralized manner. This concentration was attributed to the region’s flat terrain and the presence of extensive agricultural lands in the Chengdu–Chongqing economic circle in southwest China. Strong human activities and rapid urban development in the region led to the spread of urban living space, directly encroaching upon cropland, which served certain ecological functions, and forestland, grassland, and other ecological lands.

### Land use and carbon storage changes under various scenarios

**Figure 9:**
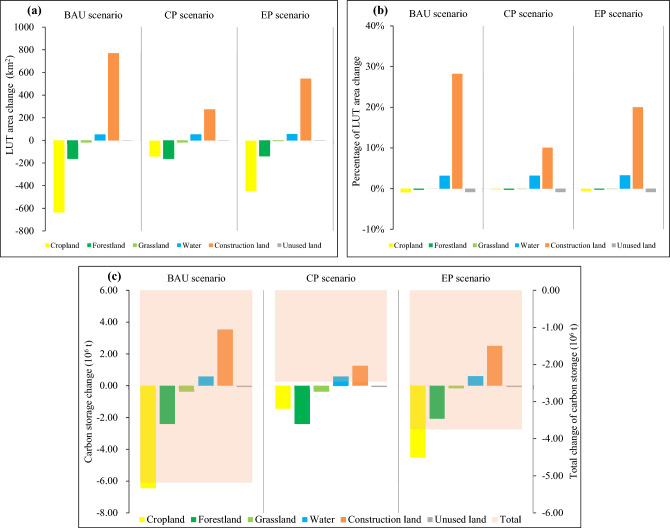
Changes in land-use area and carbon storage under various scenarios in the JRB, 2020–2035. (**a**) LUT area change; (**b**) percentage of LUT area change; (**c**) carbon storage change.

**Figure 10:**
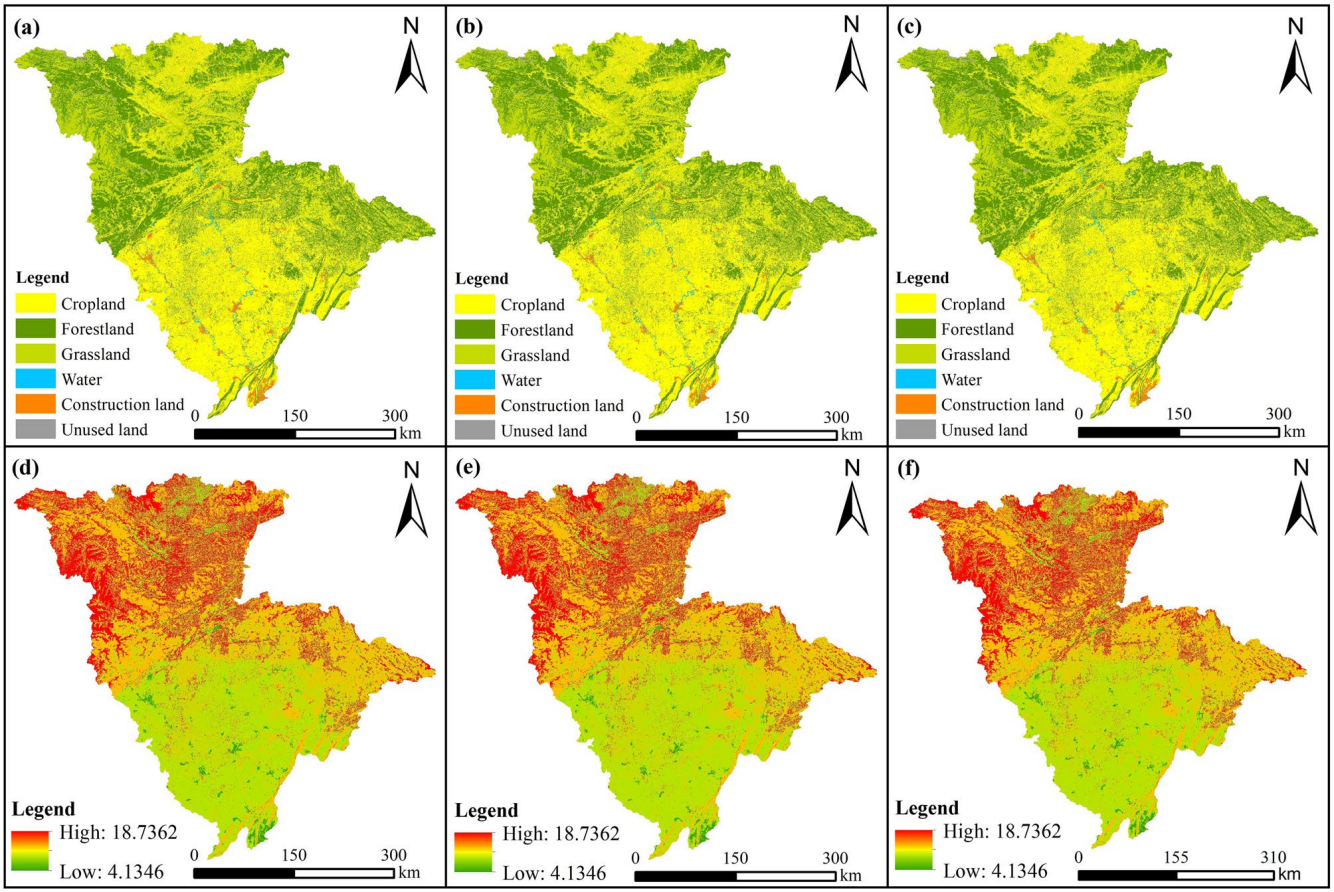
Land-use and carbon storage spatial distribution of the JRB in 2035 under various scenarios. (**a**), (**b**), and (**c**) were land-use spatial distribution under the BAU scenario, the CP scenario, and the EP scenario, respectively; (**d**), (**e**), and (**f**) were carbon storage spatial distribution under the BAU scenario, the CP scenario, and the EP scenario, respectively.

A study of land use and carbon storage in the JRB from 2020 to 2035 revealed (Figs. 9 and 10) that under different scenarios, the area of water and construction land and their carbon storage increased, while that of the remaining LUTs decreased. These changes mostly occurred in the hilly regions of the Sichuan Basin in JRB’s middle and lower reaches, and the spatial distribution of LUTs and carbon storage generally remained the same.

Under the BAU scenario, the area of construction land increased by 769.38 km^2^ (or 28.22%) over time from 2020 to 2035, while cropland, forestland, and grassland areas decreased by 637.18, 164.05, and 17.09 km^2^, or 0.91%, 0.32%, and 0.05%, respectively (Fig. 9a,b). Carbon storage reduction driven by the conversion from cropland and forestland into other LUTs was 6.44 × 10^6^ and 2.40 × 10^6^ t, respectively (Fig. 9c). Carbon storage increased by only 3.53 × 10^6^ t owing to construction land expansion, and JRB’s total carbon storage decreased by 5.18 × 10^6^ t. Without any policy constraints, construction land expansion was mainly based on the status quo of the original distribution, and construction land continued to extend along the river bank (Figs. 3e and 10a), mainly occupying cropland, forestland, and other LUTs with ecological functions to meet socioeconomic development needs. Cropland and forestland became the main areas converted into other LUTs, posing risks to food production and ecological security.

Under the CP scenario, construction land area increased by 275.21 km^2^ (or 10.09%) compared with construction land area in 2020, while cropland, forestland, and grassland area decreased by 142.46, 164.56, and 17.15 km^2^, or 0.20%, 0.32%, and 0.05%, respectively (Fig. 9a,b). Carbon storage decreased by 2.41 × 10^6^ and 1.44 × 10^6^ t owing to the loss of forestland and cropland, respectively, and increased by 1.26 × 10^6^ t owing to an increase in construction land (Fig. 9c). The total decrease in carbon storage in the JRB (2.45 × 10^6^ t) was smaller than those of other scenarios. Most basin cropland was in direct spatial competition with construction land because they were distributed close to each other (Figs. 3e and 10b). Therefore, cropland protection corresponded to constrained construction land development. Overall, the CP scenario slowed down cropland conversion while having positive effects on ecological protection.

Under the EP scenario, forestland and grassland areas decreased by 141.71 km^2^ (or 0.27%) and 7.88 km^2^ (or 0.02%), respectively, over time from 2020 to 2035 (Fig. 9a,b). Although construction land expansion was pronounced, the expansion rate decreased significantly from 28.22% (the BAU scenario) to 20.03%. Cropland area still decreased, but its decrease amount dropped from 0.91% (the BAU scenario) to 0.64%. The conversions of cropland and forestland resulted in carbon storage reductions of 4.52 × 10^6^ and 2.08 × 10^6^ t, respectively, while construction land expansion resulted in a carbon storage increase of 2.51 × 10^6^ t (Fig. 9c). Compared with the BAU scenario, the basin’s carbon storage value impairment was lower, at 3.74 × 10^6^ t. From the spatial development pattern of LUTs, the decrease in cropland area mainly occurred near construction land (Figs. 3e and 10c). In general, under the EP scenario, the conversion of ecological land such as forestland and grassland was somewhat restricted; consequently, cropland was the main type of land that experienced conversion, while reducing construction land encroachment on ecological land contributed to the preservation of the JRB ecological security.

## Discussion

### Land-use changes

This paper shows that JRB’s land-use spatial distribution is characterized by evident differentiation. Forestland and grassland are mainly found in JRB’s upper and middle reaches, while cropland and construction land are mostly distributed in JRB’s middle and lower reaches. Similar findings were reported by Xiao et al.^58^ for the Yellow River Basin (Henan section) and Wang et al.^59^ for the Taihang Mountains. The JRB spans several complex topographic and geomorphic regions, including plateaus, mountains, hills, and basins, with temperature and precipitation gradually increasing from northwest to southeast. Elevation gradually decreases as the river flows. The JRB middle and lower reaches are located in the Sichuan Basin hilly regions, where the terrain is relatively flat, facilitating agricultural and economic activities.

The study found that cropland was the only LUT that decreased in area. Previous studies have suggested that cropland was the LUT that experienced an increase, while only LUTs experienced a decrease, including forestland and unused land^1,17,41^. The main reason for this trend in the JRB, an important water source and ecological barrier in the upper reaches of the Yangtze River, is the systematic implementation of ecological fallowing measures by the government since 1999. These measures include converting cropland to forests, grasslands, and water bodies. Additionally, the development of transportation infrastructure and towns, the expansion of rural residential areas, and the continuous growth of garden land have all contributed to the reduction of cropland area.

Currently, scholars are in dispute over the results of land-use simulations under various scenarios. For example, Wei et al.^17^ estimated that in the Ebinur Lake Basin, China, cropland and construction land areas increased significantly over time from 2020 to 2030 under the BAU scenario, while forestland decreased. Conversely, under the EP scenario, cropland and construction land areas decreased, while forestland area increased considerably. Wang et al.^44^ estimated that in the Guangdong–Hong Kong–Macao Greater Bay Area, under the BAU scenario, cropland and forestland areas decreased over time from 2020 to 2030, while construction land and grassland areas increased, and under the CP scenario, cropland, grassland, and construction land areas increased. Yang et al.^43^ estimated that in Xi’an City, China, under the EP scenario, cropland area decreased over time from 2015 to 2030, while construction land, forestland, and grassland areas increased, and under the CP scenario, construction land area increased, while cropland, forestland, and grassland areas decreased. Our study results show that cropland, forestland, and grassland areas decreased under different scenarios, while construction land area increased significantly, with the smallest amplification in construction land under the CP scenario. A comparison of these studies reveals differences in policies related to territorial spatial planning, economic and social development, and ecological conservation in various study areas. These differences affect the setting of land-use transfer probabilities. Substantial variations in the initial land-use patterns and land-use transfer probabilities in the different study areas lead to differences in land-use simulation results under different scenarios.

### Carbon storage changes

Carbon storage in the JRB initially increased and then decreased over time from 2000 to 2020, a trend that aligns with the findings of Gong et al.^4^ and Chen et al.^60^. This pattern is primarily attributed to the policy implementation of returning farmland to forests since 2003, resulting in the conversion of cropland to forestland and an increase in carbon storage. However, as urbanization accelerates, rural populations migrate to towns and cities, leading to the expansion of urban boundaries and the constant encroachment into ecologically functional lands around towns and cities. The urban boundaries encroach into a large area of cropland and small areas of forestland and grassland. This ultimately results in a continuous decline in carbon storage.

Our study results show that JRB’s carbon storage was aggregated and generally increased over time; however, the JRB featured no “high–low” or “low–high” clustering region, whereas scholars^32,33,61,62^ reported “high–low” and “low–high” clustering patterns for different study regions. Carbon storage high-value and low-value clustering reveal clear distribution boundaries between cold spots and hot spots according to their spatial distribution pattern. These findings differ from those of previous research; for example, Li et al.^33^ reported spatial aggregation of carbon storage in Kunming City with fluctuating changes over time. Liang et al.^7^ found that cold spots and hot spots in carbon storage on the Loess Plateau represented a small percentage of the total. The cold spots and hot spots exhibited a mosaic and decentralized distribution^7,33^. Lin et al.^61^ found that the spatial distribution of carbon storage in Guangdong exhibited clustering phenomena, with the degree of agglomeration initially increasing and then decreasing over time. However, the distribution of cold spots and hot spots was scattered, and distinct demarcation lines were lacking. The topography in the northwest of the region is higher, while the southeast of JRB features lower terrain. In the upper reaches of the Jialing River, the landscape consists of meandering courses with deep valleys, whereas the middle reaches exhibit flatter terrain, transitioning from deep hilly regions to shallower hilly areas. In the lower reaches, the main river runs parallel to the eastern part of the Sichuan Basin, forming canyon extensions. The lower basin rises to mountainous terrain. Consequently, the middle and lower reaches of the JRB are characterized by frequent human activities, primarily cropland, leading to lower and patchy carbon storage distribution. In contrast, the topography in the middle and upper reaches of the JRB is dominated by mountainous terrain, including some plateau landforms. These areas feature higher elevations and lower temperatures and precipitation, resulting in a landscape dominated by forestland and grassland. The distribution of forestland and grassland is mosaic-like, which prevents the concentration of high carbon storage regions in a patchy manner. The marginal mountain region of the Sichuan Basin forms a strongly ascending fold belt with significant relative elevation differences. This region exhibits a clear transition from the hilly areas of the JRB, leading to a distinct boundary between high and low carbon storage value distributions.

### Impact of land-use changes on carbon storage dynamics

From 2000 to 2020, the change in carbon storage caused by the conversion of cropland to other LUTs accounted for 71.70% of the total change in carbon storage in the JRB. The reduction in carbon storage due to the conversion of various LUTs to construction land was 1.72 times the total reduction in carbon storage in the JRB. Specifically, the reduction of carbon storage caused by the conversion of cropland to construction land was 1.45 times the total reduction in carbon storage in the JRB. Therefore, the conversion of cropland to construction land between 2000 and 2020 was the main factor driving the reduction in carbon storage in the JRB. This finding is consistent with the results of Ren et al.^63^ on the impact of land-use change on carbon storage in Gansu Province. However, Zhang et al.^64^ found that the conversion of cropland to other LUTs generally led to an increase in carbon storage. In the JRB, carbon storage decreased owing to the following two factors: (1) cropland area experienced a net loss, as 61.11% of the converted cropland area was transformed into construction land; (2) the total carbon density of construction land was only 45.45% of that of cropland.

Studies have yielded varying results regarding the impact of land-use changes on carbon storage under different scenarios. For instance, Li et al.^45^ demonstrated that in the northeastern part of the Tibetan Plateau, carbon storage decreased over time from 2020 to 2030 under the BAU scenario, and the carbon storage increase in the EP scenario exceeded that of the CP scenario. In a study of Changchun City conducted by Li et al.^19^, carbon storage was projected to decrease over time from 2020 to 2030 under the BAU scenario, with less value impairment in the CP scenario compared with the BAU scenario, and an increase was observed under the EP scenario. Liu et al.^48^ found that carbon storage increased over time from 2020 to 2035 under the BAU, CP, and EP scenarios in the Loess Plateau. In contrast, carbon storage in the JRB decreased under different scenarios, with the least value impairment occurring under the CP scenario and the most significant value impairment occurring under the BAU scenario. An examination of historical land-use changes in the JRB reveals that most of the newly added construction land originated from cropland, resulting in intense competition between these two land types. Construction land area increased substantially in different scenarios, while forestland and cropland with ecological functions decreased significantly. This directly leads to a decrease in carbon storage under different scenarios. The implementation of CP policies minimizes the encroachment of cropland and, consequently, reduces construction land expansion. Therefore, in the future, following the Chinese government’s requirements for replenishing cropland, the JRB should increase the cropland area through the reclamation and remediation of unused land suitable for cultivation and the construction of high-standard farmland. This will enhance the carbon sequestration function of the JRB ecosystem, thereby ensuring food and ecological security in the region and the realization of the “dual-carbon” goals.

### Limitations and directions for future work

The three future scenarios for the JRB presented in this paper, established using Markov and PLUS models, may not comprehensively represent all potential future land-use scenarios for the region. Given that JRB encompasses various topographic and geomorphic zones, including plateaus, mountains, hills, and basins, there exist significant disparities in both natural environments and human geography. Therefore, future studies should consider dividing the JRB into distinct regions according to topography and geomorphology, allowing for more precise investigations of land-use changes and carbon storage estimations in each region. Additionally, the study identified 19 driving factors solely for simulating future scenarios, without analyzing in detail the driving forces behind land-use changes in the JRB. To comprehensively elucidate these factors, their impact on land-use changes should be further explored through methods such as geodetector models. Moreover, although this paper elucidates the changing patterns of land use in the JRB, it does not extensively explore the competitive relationships between various LUTs and the strategic decisions in the development and utilization of land resources. Therefore, future research should comprehensively analyze land-use conflicts within the JRB.

## Conclusions

This paper explored the evolving dynamics of land use and carbon storage within the JRB between 2000 and 2020. Utilizing land-use data from 2020 as a foundation, a coupled PLUS–InVEST model was applied to simulate and predict land-use and carbon storage patterns for 2035 under varying scenarios. The study also investigated the ramifications of land-use alterations on carbon storage, resulting in the following key findings and principal conclusions:

1. The PLUS–InVEST coupled model demonstrated strong applicability within the JRB. Model validation yielded an OA of 0.9961, and an FoM coefficient of 0.0232, signifying a high level of simulation accuracy. These results demonstrate the PLUS–InVEST model effectiveness in forecasting future land-use patterns within the JRB.
2. Cropland was the predominant LUT in the JRB, encompassing over 43% of the total land area. It was primarily distributed in the middle and lower reaches of JRB, specifically within the hilly regions of the Sichuan Basin. Moreover, cropland was the only LUT that experienced a decrease in area; the decrease amounted to 1,673.27 km^2^, of which 61.11% was converted into construction land. This indicates the existence of a contradiction between human needs and available land resources in the JRB. This situation necessitates the scientific formulation of land-use policies in the JRB and calls for the exploration of new pathways for the coordinated development of regional urbanization and food and ecological security.
3. The three most substantial carbon reservoirs in the JRB—cropland, forestland, and grassland—collectively accounted for over 98% of the total carbon storage in 2000–2020, a sum which decreased over time. The conversion of cropland to construction land between 2000 and 2020 was the primary factor driving the reduction in carbon storage in the JRB. This resulted in an overall weakening of the carbon sequestration capacity of the ecosystem in the JRB. Carbon storage exhibits a clear spatial aggregation and stabilization within the JRB, with higher levels observed in the northern regions and lower levels in the southern areas. Potential strategies to address these trends and promote ecological security include implementing more afforestation and grass-planting initiatives in the middle and upper reaches of the JRB. Additionally, efforts to decelerate urbanization in the middle and lower reaches of the JRB could mitigate ecological risks.
4. Across different scenarios, there was a consistent pattern of increasing water and construction land areas, along with corresponding carbon storage expansion. Conversely, the areas and carbon storage of other LUTs decreased over time from 2020 to 2035, with these shifts predominantly occurring in the middle and lower reaches of the JRB. An examination of the period from 2020 to 2035 under the CP scenario revealed that cropland area exhibited a minimal decline of only 0.20%, significantly less than the 0.91% decrease in the BAU scenario and the 0.64% decrease in the EP scenario. Moreover, the CP-scenario carbon storage value impairment amounted to 2.45 × 10^6^ t, which was 47.42% of the carbon storage value impairment in the BAU scenario and 65.56% of that in the EP scenario. Therefore, under the scenarios outlined in this paper, although the CP policy can effectively control the rate of cropland area reduction, it cannot prevent the reduction of cropland area, and the carbon sequestration function of the ecosystem in the JRB will still be weakened. Therefore, strict adherence to the task of replenishing cropland mandated by the Chinese government is imperative. Increasing cropland area through land remediation and high-standard farmland construction is crucial for enhancing the carbon sequestration function of the JRB ecosystem. This approach will positively contribute to ensuring the food and ecological security of the JRB and achieving the “dual-carbon” goals.

## Data Availability

All data presented in this study are available from the corresponding author on reasonable request.
